# A national survey of the radiotherapy dosimetrist workforce in the UK

**DOI:** 10.1259/bjr.20220459

**Published:** 2022-09-21

**Authors:** Nicola Blackler, Karen E Bradley, Charles Kelly, Steven Murphy, Carole Cross, Mike Kirby

**Affiliations:** 1 Plymouth Hospital NHS Trust, Plymouth, UK; 2 North West Cancer Centre, Londonderry, Northern Ireland, UK; 3 Northern Centre Cancer Care, Newcastle, UK; 4 The Christie NHS Foundation Trust, Manchester, UK; 5 The British Institute of Radiology, London, UK; 6 The University of Liverpool, Liverpool, UK

## Abstract

**Objectives:**

To undertake a national survey of the Radiotherapy Dosimetrist workforce within the UK; examining different attributes and experiences, comparing results with published evidence within the literature.

**Methods:**

A national, anonymised survey was undertaken between Dec 2020 and end of Feb 2021; employing a mixed-methods approach and blend of closed, open-ended answer choices and free-text comments. Questions included range of training routes and job titles; registration status; job tasks and engagement with Continuing Professional Development (CPD).

**Results:**

A total of 223 individuals responded. Nearly half were trained via therapeutic radiography; approximately, a fifth through a clinical technologist/physics routes. Most (70%) had Dosimetrist in their job title. Nearly 70% were statutorily registered, and almost a fifth were in the voluntary register of Clinical Technologists. Most job tasks were in treatment planning – with 57% spending over 70% of their time there. Most notably, 29% were not involved in any CPD scheme. No published evidence showed the same aspects identified here.

**Conclusions:**

Our survey showed a unique profile of the Radiotherapy Dosimetrist workforce in the UK, with a variety of training routes and statutory registration status. Nearly, a third were not engaged in a CPD scheme – adding to the current discussion that perhaps all Dosimetrists should be statutorily registered, for ensuring safe and effective clinical practice.

**Advances in knowledge:**

A novel and unique national survey of Dosimetrists working in Radiotherapy in the UK is presented, leading to new insights into current training routes, registration status, job tasks and CPD engagement and needs.

## Introduction and background

The dosimetrist workforce has been an integral part of the team of professionals working in radiotherapy within the UK for many years.^1^ Often their training has come through clinical technologists training through the Institute of Physics and Engineering in Medicine (IPEM) or as Therapeutic Radiographers with a well-defined, Health and Care Professions Council (HCPC) statutory registered pathway.^2–5^ Those electing for the route through IPEM are encouraged to join, voluntarily, the register of Clinical Technologists as accredited by the Professional Standards Authority.^4–6^ But, like other professionals who also work within Radiotherapy (such as Linac engineers), and indeed some other disciplines within the NHS, they are not required to have statutory HCPC registration or its equivalent for their jobs.

In some countries, the role, profession and career pathway are much more structured and robust for Dosimetrists. For example, in the USA, where dosimetrists have their own professional body (the American Association of Medical Dosimetrists),^7^ their own specific, peer-reviewed scientific journal (Medical Dosimetry)^8^ and the encouragement to also have board certification through the Medical Dosimetrists Certification Board,^9^ although this is still not a mandatory requirement nationally. Having such a structure provides Dosimetrists with greater autonomy and professional standing; with the workforce regularly surveyed in terms of their opinions and staffing levels.^10–12^

Within Europe, surveys through the Health Economics in Radiation Oncology (HERO) project have examined staffing levels for dosimetrists, showing a wide variation in their employment^13,14^; although a key confounding variable within the Europe-wide surveillance is the disparity in demographic indicators – countries employing a mix of staff employed in different roles throughout the radiotherapy pathway.^13,14^ For example, some countries do not have recognised dosimetrists or do not use the terminology; or other staff groups (*e.g.,* Radiation Therapy Technologists (RTTs) – in the UK, Therapeutic Radiographers) undertake planning responsibilities, but are not referred to as dosimetrists. There are countries where nurses operate treatment machines, and therefore are not classed as RTTs.^13,14^

The role of the dosimetrist is a vital one within radiotherapy, primarily involved with and responsible for the production of highly accurate and precise computerised treatment plans for patients’ treatments.^2,7^ Working together with clinical oncologists and radiotherapy physicists, these range from the straight-forward to the most complex state-of-the-art plans for X-ray and particle therapy. They may also be involved with areas outside of treatment planning, with radiation dose measurements and equipment quality assurance.^2,7^

However, to our knowledge, no survey has been conducted and published examining the work, training, registration and CPD profile of UK dosimetrists. Our survey here (undertaken through a small working party from the management committee of British Institute of Radiology’s (BIR’s) Radiotherapy and Oncology Special Interest Group) is in response to that; to fill that hole in the published evidence by examining a wide range of attributes from the Dosimetrist workforce – their range of training routes and qualifications, registration status, the tasks they undertake within Radiotherapy, their professional development requirements etc.. These are aspects not covered in the comprehensive ESTRO-HERO project when examining workforce resources^13,14^; nor in the most recent works which have mainly modelled time-driven activity-based costings,^15^ reimbursement^16^ and developing a value-based framework for innovation and technology assessment.^17^

To compare and contrast with published literature, our results were evaluated in light of any other similar surveys undertaken; these were identified through a structured, systematic search of PubMed focusing on Dosimetrists.

For brevity, only the key, quantitative results from our survey are presented here; the detailed qualitative and free-text comments will be covered in a follow-on publication.

## Methods

A survey was designed using the Survey Monkey platform, as used in previous questionnaires undertaken by groups within the BIR. A mixed methods approach was adopted with a series of closed and open-ended answer choices for the majority of questions; and numerous opportunities for free text comments. All responses were completely anonymised and full GDPR controls were undertaken, with no identifiable data being visible within any of the analysis. The link to the survey was emailed out through many different channels including; the BIR’s own membership; through professional links with IPEM, the Royal College of Radiologists (RCR) and College of Radiographers (CoR); through closed email contact lists for Heads of Radiotherapy and Radiotherapy Physics; and through the Medical Physics Mailbase. The survey was conducted between Dec 2020 and end Feb 2021. The questions asked are shown in Table 1.

**Table 1.** A resume of the questions posed in the survey

Question posed:	Answer Choices
What training route did you initially follow?	HNC/HND MPPM [2year Block Release] + Clinical portfolio ONC MPPM [2year Block Release] + Clinical portfolio Degree course in BSc (Hons) Clinical Technology+IPEM Clinical Tech Diploma [4year Block Release] Graduate Dosimetrist scheme+IPEM Clinical Tech Diploma [2year Block Release] Degree course in BSc (Hons) Clinical Technology [3year Fulltime with Clinical Placements] Diploma in Therapeutic Radiography [2years] Degree course in BSc (Hons) Therapeutic Radiography [3year Fulltime with Clinical Placements] Degree (non-allied)+on the job training Equivalence routes allied BSc or MSc degree+Clinical training In-house training, please add details of pathways/level of qualifications Other (please specify)
What is your current job description title?	Radiographer Clinical Technologist Healthcare Science Practitioner Dosimetrist Other (please specify)
What registers, professional bodies or organisations are you currently a member of?	HCPC IPEM CT IPEM RSci IPEM CSci IOP Academy of HCS BIR SCoR ESTRO AAPM Other (please specify)
If you could change you job title, what title would you prefer to use?	Radiographer Clinical technologist Healthcare scientist Dosimetrist Happy with current title Other (please specify)
If you wish to change your job title, why do you?	- Free text comments
What tasks do you do in your job? Rate the percentage time spent	Treatment Planning Machine QA Mould Room Patient specific QA *In vivo* dosimetry Brachytherapy Specialist planning (SRS/SABR/Proton)
Are you currently a member of a CPD scheme?	IPEM HCPC CPD Now Register of Clinical Technologists (RCT) I am not a member of a CPD scheme Other (please specify)
What are your CPD needs?	Basic Physics modules Physics refresher modules Basic anatomy Anatomy refresher modules Advanced anatomy modules Other (please specify)
If you would like refresher sessions, what topics would you like to see?	- Free text comments
What difficulties have you experienced in your training in regard to accessing appropriate training content?	Funding Lack of time I haven’t experienced any difficulties in accessing training Free text comments
Opportunities for Public Engagement: how likely would you be to engage in?	1–2 h online shared practice, hot topic discussion VMAT/RapidArc Breast Planning Sharing TPS approaches and software tricks (Site specific) Radiotherapy Planning OAR outlining (Site specific) ‘Conquering Contouring’ - Manual and Atlas based contouring, for OAR’s & Volume definition Insight into Proton Planning The basics of IGRT: what do we want, how much imaging should we use? What do others do? Abdominal compression, what’s out there, shared experiences Workflow management – paperless working Scripting Remote working/Planning. Connectivity of IT Systems: Citrix. What are the options out there?
How often would you like to engage?	Quarterly hot topics Six monthly Annually On-demand Other (please specify)
What other subjects would you like to see covered?	- Free text comments
What would be your choice of delivery format?	Short on-line Webinars Face-to-face Online taught and assessed modules Other (please specify)
When is the best time for you to complete your CPD?	Early morning Lunchtime Afternoon Evenings Saturdays

This type of convenience sampling approach compares well with the most recent professional surveys from IPEM,^18^ RCR^19^ and CoR^20^ which sought data only from the Heads of Radiotherapy Physics, the Clinical Leads and the Heads of Radiotherapy in each Cancer Centre. But in contrast, to go beyond primarily workforce resource data from service leads, our survey sought to gather wider information (likely not held or readily available to service leads) and opinions and experiences of individuals working as dosimetrists within the UK. This generalised, non-targeted method has advantages and disadvantages, as discussed later in the *Limitations* section.

Three separate structured literature searches were undertaken through PubMed, using the search terms of “dosimetrist AND (survey or questionnaire)”; “dosimetrist professional development” and “dosimetrist survey”. The inclusion and exclusion criteria detailed in Table 2 were then applied to produce a final list of papers for critical comparison

**Table 2.** Inclusion and Exclusion Criteria used for the structured literature searches

Inclusion	Exclusion
Primary Data sources Texts in English Peer-reviewed sources UK and non-UK sources Papers highlighting aspects of registration/certification, job tasks, CPD, education, training pathways and formats for dosimetrists Surveys of dosimetrist workforces in other aspects	Sources which are commentaries, editorials, reviews or web-based articles Texts not in English Sources which are not peer-reviewed Sources published before 2000 Sources which are not accessible as full-text

## Results and discussion

Two hundred and twenty-three responses were received over the data collection period. In short, the data showed dosimetrists have a wide range of training routes/qualifications; most had dosimetrist within their job title and were happy with their overall job title; the majority had HCPC registration and, as one might expect, their most active area of involvement was in treatment planning; most respondents listed HCPC and the Register of Clinical Technologists (RCT) for membership of a Continuing Professional Development (CPD) scheme; advanced anatomy and basic Physics featured most in terms of CPD needs, although the majority also noted a lack of time as a barrier to accessing appropriate training; the workforce showed great forward-thinking in still being willing to engage publicly in their work through shared learning and other methods; in terms of CPD and engagement format, over two-thirds favoured the use of webinars, with a willingness to engage in activities in evenings and weekends.

The recent report from the CoR^20^ noted that there were 321 therapeutic radiographers working in dosimetry – an average of 5.3 in each radiotherapy provider. IPEM^18^ reported in their recent survey that there were 565 in post WTE (Physics) clinical technologists. The data for both these surveys was gathered through individual Heads of Service. Our responses, from individual dosimetrists, would therefore suggest a good completion rate of nearly 40%. Also, in comparison, the IPEM,^18^ RCR^19^ and CoR^20^ surveys focussed on workforce resource data in terms of establishment and vacancy profiles, job bandings, absence rates, expected retirement rates, as well as heads of service opinions on morale, stress and burnout.^18–20^ It is clear that our survey has focused on different, but complementary, characteristics for dosimetrists. There is scope to examine similar resource characteristics, specific to dosimetrists, in future surveys (see *Futures* section).

### Training routes

**Figure 1.**
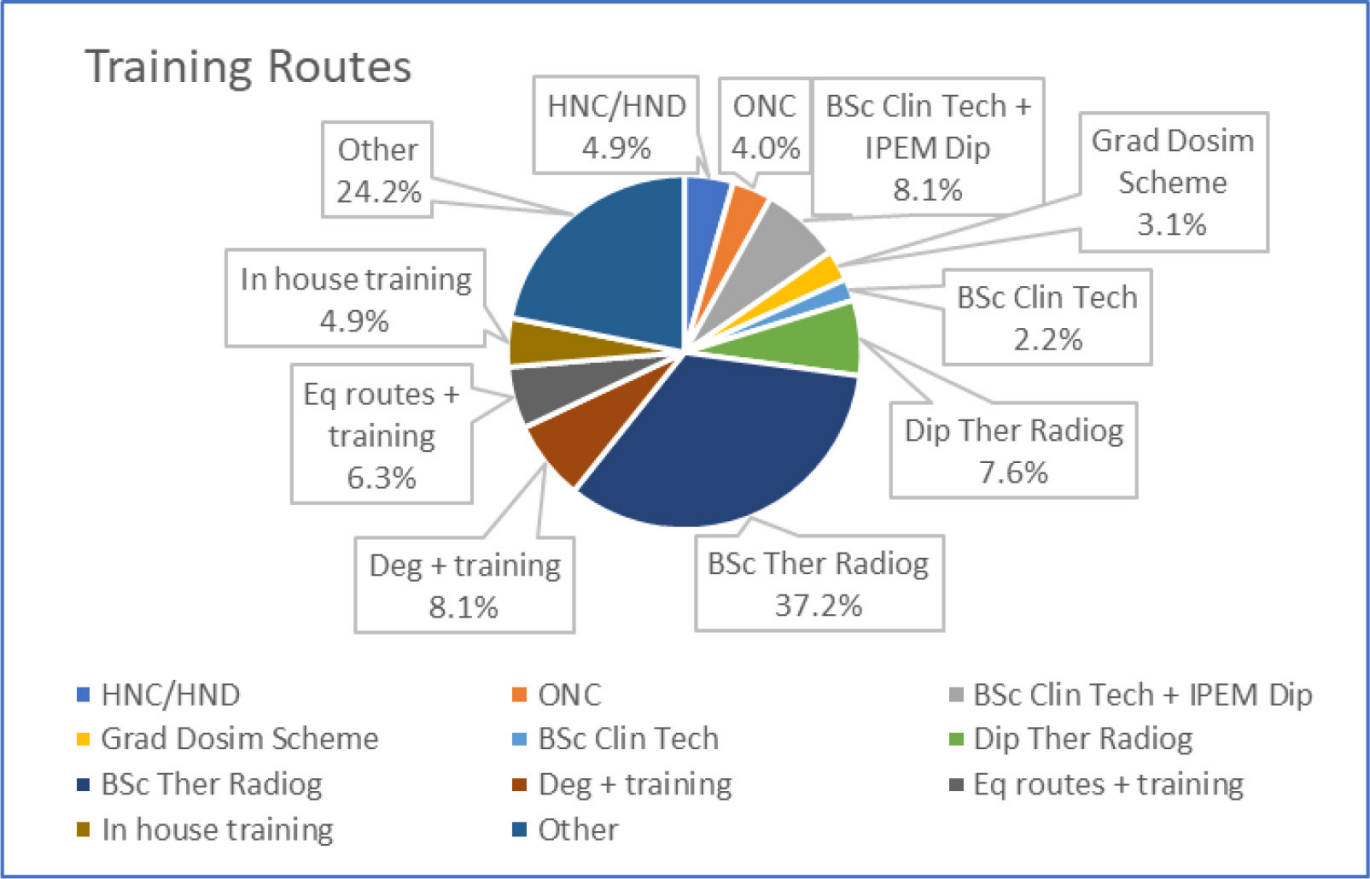
The distribution of training routes for the dosimetrist respondents

The distribution of declared training routes are shown in Figure 1 and detailed in Table 3. From the closed question choices, nearly 45% listed a training route which was based on therapeutic radiography – either through a 2 year or 3 year course. Nearly 14% were engaged through a clinical technologist training route, of which most undertook a 4 year block release course. Fifty-four people added detail under the ‘other’ category; of which 17% identified a Physics based degree; 33% a therapeutic radiography degree/PG diploma; 33% with another degree and 17% coming in via another route.

**Table 3.** Further detail of the variety of training routes for the dosimetrist respondents

Answer Choices	Percentage Responses
HNC/HND MPPM (2year block release) + Clin Portfolio ONC MPPM (2year block release) + Clin Portfolio BSc Clin Tech + IPEM Clin Tech Dipl (4year block release) Grad Dosimetrist Scheme + IPEM Clin Tech Dipl (2year block release) BSc Clin Tech (3year fulltime+Clin placements) Dipl Therapeutic Radiography (2years) BSc Therapeutic Radiography (3year fulltime+Clin placements) Degree (non-allied)+job training Equivalence Routes allied BSc or MSc + Clin training In-house training Other	4.9% 4.0% 8.1% 3.1% 2.2% 7.6% 37.2% 8.1% 6.3% 4.9% 24.2%

### Job title, registration and professional bodies

Of the respondents, sixty-six percent had dosimetrist within their job title and 14% had clinical technologist. Twenty-two respondents qualified their responses further with other choices, emphasising their titles included prefixes like senior, lead or principal dosimetrist (six). For those with technician or technologist (seven), further prefixes included senior, advanced, principal and head. Five listed that they were a practitioner or radiographer.

Sixty-four percent of respondents were happy with their current job title; but 22% (49) wished to have dosimetrist in their job title. Fifteen respondents clarified further their opinions, with four wishing to have ‘treatment planner’ or ‘radiotherapy treatment planner’ in their title; or ‘radiotherapy/medical dosimetrist’.

**Figure 2.**
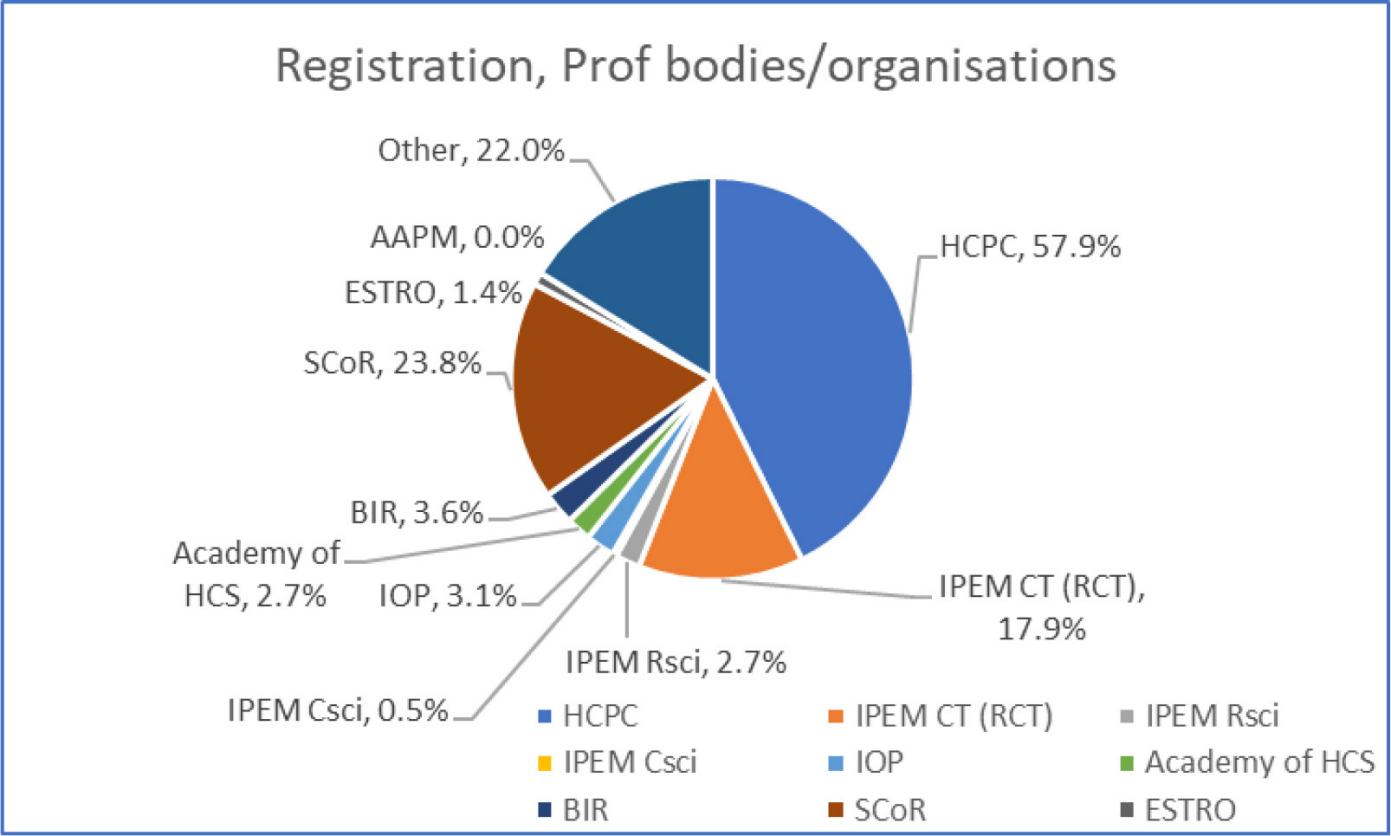
The proportions of respondents indicating registration and membership of professional bodies/organisations

In terms of registration, membership of professional bodies and organisations, the distribution of replies is shown in Figure 2 and detailed in Table 4. Nearly sixty percent of the respondents listed HCPC registration; 24% listed membership of SCoR and 18% membership of IPEM as a clinical technologist. Forty-nine respondents qualified their answers further, with 29 of the 49 (59%) noting their membership of the voluntary Register of Clinical Technologists or applying for that. Twelve of the 49 (25%) noted that they were not registered.

**Table 4.** Further detail of the responses regarding registration and professional body/organisation membership

Answer Choices	Percentage Responses
HCPC IPEM CT IPEM RSci IPEM CSci IoP Academy of HCS BIR SCoR ESTRO AAPM Other	57.9% 17.9% 2.7% 0.5% 3.1% 2.7% 3.6% 23.8% 1.4% 0% 22.0%

### Job tasks

**Figure 3.**
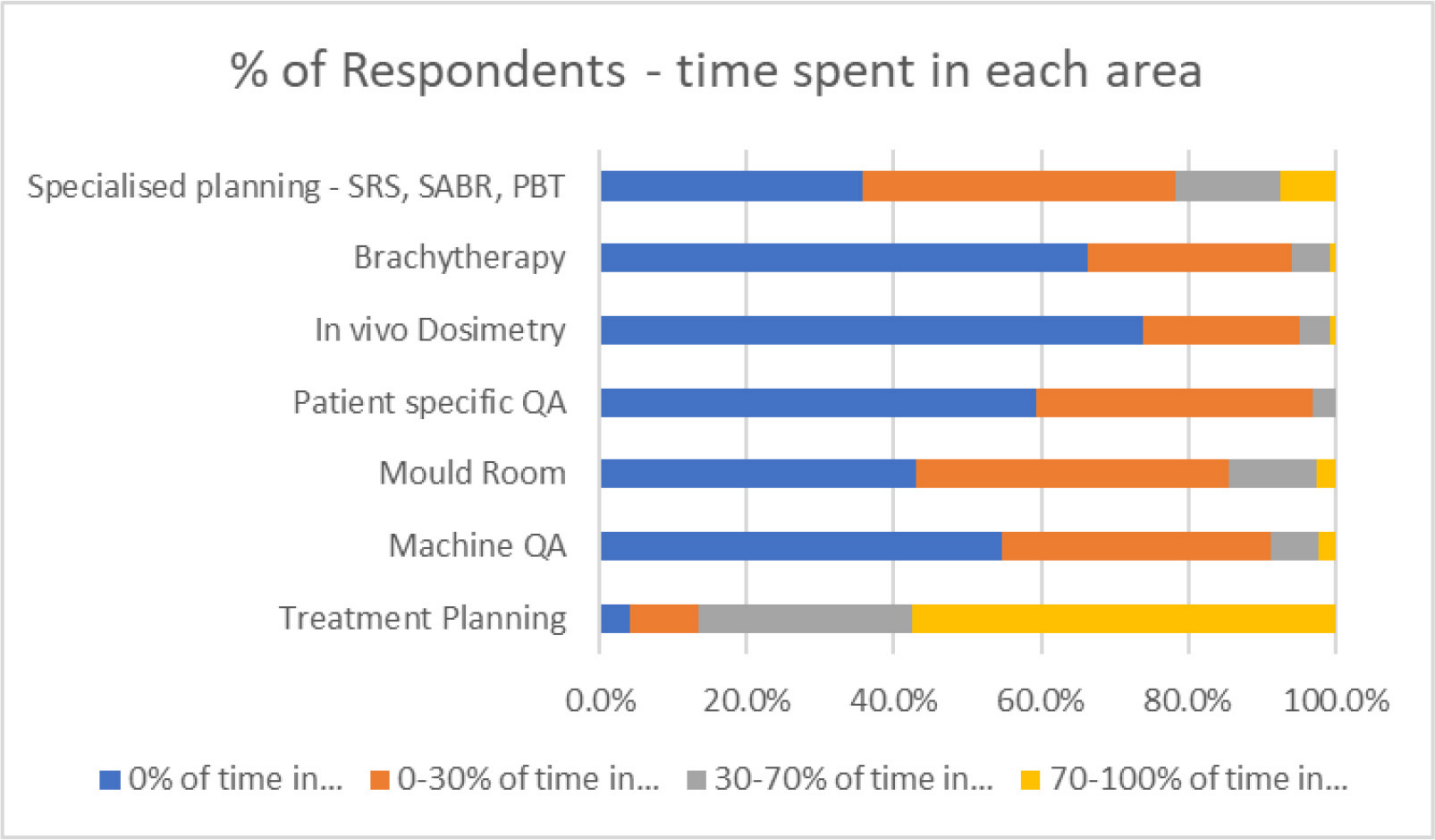
The data from Table 5 illustrated as a stacked bar chart

By far the majority of tasks was that of treatment planning (Table 5). One hundred and twenty eight of the 223 respondents (57%) indicated that they spent 70% or more of their time on treatment planning. Only nine respondents (4%) noted they did not spend any time on treatment planning, indicating that some dosimetrists’ work is entirely outside computerised planning. The proportions for the other specialties are shown in Figure 3; showing that between a fifth and two-fifths of the respondents in each category had some other duties (such as machine QA, mould room duties and patient specific QA) within their normal range of tasks, up to 30% of their working time. It should be noted that there were fewer respondents for the categories other than treatment planning (range 123–162). Seven percent (of 162 respondents) noted that they were involved in specialist planning (*e.g.,* SRS/SABR/Proton planning) for more than 70% of their time.

**Table 5.** Percentage of respondents spending the indicated % working time in each specialty

% Time spent in….	Treatment Planning	Machine QA	Mould Room	Patient specific QA	*In vivo* Dosimetry	Brachytherapy	Specialised planning - SRS, SABR, PBT
**0% of time in…**	4.0%	54.7%	43.1%	59.3%	74.0%	66.4%	35.8%
**0–30% of time in…**	9.4%	36.5%	42.5%	37.8%	21.1%	27.6%	42.6%
**30–70% of time in…**	29.1%	6.6%	11.8%	3.0%	4.1%	5.2%	14.2%
**70–100% of time in…**	57.4%	2.2%	2.6%	0.0%	0.8%	0.7%	7.4%
Respondents	223	137	153	135	123	134	162

From some of the qualitative comments received, also identified as key tasks were administration; management of staff; meetings and training of staff; R&D projects; maintaining the quality system; advice for clinical set-ups on-set and involvement in other specialised areas not identified in the question.

### Continuing Professional Development (CPD)

Eighty percent of respondents (179/223) indicated they were involved in CPD schemes, through HCPC, IPEM, CPD Now and the Register of Clinical Technologists. Strikingly, 29% indicated that they were not a member of a scheme. In terms of considering CPD needs, 52% wished to engage with advanced anatomy modules; 49% with Physics refresher modules; 41% with advanced Physics modules and 40% with anatomy refresher ones.

In line with some of the other job tasks identified earlier, some free-text comments indicated a need for training and development in management, leadership and coaching/teaching, project leadership and development alongside the scientific topics identified of advanced techniques and latest developments in radiotherapy.

In terms of challenges to undertaking CPD and training, 37% (83) identified funding as one the key difficulties, and 73% (163) noted a lack of time being a key point. Twenty-two percent (49) indicated that they haven’t experienced any difficulties in accessing training. A number of free-text comments voiced a lack of time, accessibility and availability of appropriate courses (online), with some noting that support from managers can be challenging, with little identifiable career development structure to engage in.

### Opportunities for engaging publicly

With hindsight, this survey question could have been less ambiguous; but it provided further detail on engagement with the wider community for CPD through shared learning etc.. Despite four-fifths of respondents indicating challenges to undertaking training and CPD, averages of 28 and 17% of respondents respectively were either very likely or definitely willing to engage in the proposed categories. The top three were; 31% (69 out of 221 respondents) definitely wishing to discuss VMAT breast planning; 26.5% (59 out of 223 respondents) to discuss manual and atlas based site-specific contouring for OARs and volume definition; 25% (55 out of 219 respondents) to share TPS approaches and software tricks for different clinical sites.

Most (50%) wished to do so on quarterly basis, focusing on hot-topics and 26% were willing to do this on-demand.

### Delivery format and timing

In terms of format, for delivery of such topics, and likely generally engaging with CPD, there appeared to be a range of formats of delivery respondents would choose. Most (over two-thirds, 69%, 153 respondents) chose webinars; 57% (128) chose short, online formats; 50% chose online taught and assessed modules and 25% wished the delivery to be face-to-face. Thirteen offered further comments through the Other choice – some looking for a range of formats, noting that it would depend upon the subject matter and some highlighting group, workshop and hands-on style formats.

In terms of timing many were willing to engage in such activities outside what might be described as core time – *i.e*. evenings and weekends. Forty-four percent favoured afternoon activities; 37% evenings; 31% early morning; 29% lunchtime and 19% Saturdays.

### Published literature

From the searches conducted through PubMed, a total of 85 papers were identified, ranging in publication date from 2021 to 1989. Thirty-two were chosen for relevance (through the inclusion/exclusion criteria detailed in Table 2) and comparative discussion with findings from our research work here.

By far the majority of papers were from the US (22); there were five from Canada, two from Australia/New Zealand, one from Ireland (which covered a worldwide survey) and two from Europe as part of the HERO initiative.

None were identified covering the exact same aspects as we did in total with our survey. Types of survey and issues examined are shown in Table 6 .

**Table 6.** Key themes and issues examined from published literature extracted from the systematic searches

Some of the issues surveyed and examined	References
Whole radiation oncology workforce demographics, salaries, challenges, opportunities, attitudes to supply, staffing models (US, Canada, Australia, New Zealand and Europe)	10, 11, 12, 13, 14, 21, 22, 23, 24, 25
Confidence, proficiency, training needs, comparisons of training routes (*e.g.,* RTT trained cf non-RTT trained) (US)	26, 27
Attitudes and responses to flexible and innovative working (*e.g.,* remote working in response to Covid) (US)	28, 29
Proficiency and expertise for tasks compared with other staff groups (US)	30
Attitudes and contributions towards research and technique/service development (US and Canada)	31, 32, 33
Utilising experience and expertise of dosimetrists as valued team members within Radiotherapy; for routine and specialised treatments; in education and training (for workforce and others) and taking part in research (US and Canada)	21, 32, 33, 34, 35, 36, 37, 38, 39
Contributions, views, opinions, comfort and expertise towards patient safety, incident learning platforms (US, Canada, Ireland/worldwide)	35, 40, 41, 42, 43, 44, 45
Issues and health experiences of the dosimetrist workforce (US)	46, 47

Within the papers identified were key workforce studies, like our own; but primarily examining the staffing resource (demographics, capacity, staffing levels and models etc.) of dosimetrists within radiation therapy, sometimes as part of surveys of the whole radiation therapy workforce.^10–14,21–23^ Surveys ranged in number from a few tens to thousands; where the number of dosimetrists involved were from a few tens to over two thousand for some of the professional studies of the dosimetrist workforce alone in the US.^10,24–27^ Our study, at 223 respondents, is reasonably placed within these and also likely a good representation of the UK dosimetrist workforce; the European studies of workforce and capacity highlight a total of 43 dosimetrists, but for Wales and Northern Ireland only – there were no applicable numbers for England and Scotland.^13,14^ As mentioned earlier, a key difficulty is identifying job titles matching the key roles involved across many different countries in Europe – where the tasks of dosimetrists, clinical technologists and therapeutic radiographers in the UK, are undertaken by staff with different titles and different clinical professions too in other countries in Europe.^13^ Here we found a similar landscape, with nearly 70% of our respondents having “Dosimetrist” within their job title, whilst others had a variety of “Radiographer”, “Clinical Technologist” or “Healthcare Science/Physics Practitioner” terms used. Some surveys in the literature used Survey Monkey^21,22,28^ or Qualtrics^24^ as platforms, in a similar manner to our study; some were distributed only through professional body or board certification membership, unlike ours.^10,24–27,29^

A common theme in much of the literature examined is the value of the dosimetrist in the radiotherapy workforce; how important and integral their role is to a modern, efficient and effective radiotherapy service.^29–37^ They are professionals with high expertise,^29,32,33,35,36^ can exercise flexibility (and willingness) for different ways of working *e.g*. remotely within the Covid-19 pandemic, whilst still maintaining a high-quality service.^30,31^ Their expertise and experience are valued in contributing to patient safety^28,37–40^ and incident learning systems.^25,39,41^ Their expertise helps develop and shape radiotherapy practices and techniques,^30,33,38^ contributing to and being involved with research^29,33,42^ and subsequent training of other staff groups.^29,43^ They have a key role in education and training, often ideally within a multidisciplinary environment through interprofessional learning/education (IPE/IPL), in new and innovative ways.^24,29,35,43–45^

Some key similarities can be identified with the results of our own survey. There are various training routes and aspects of certification (registration within the UK) for the dosimetrist workforce^46^; with some models (like the US) being more established and well-recognised as a distinct professional workforce within radiotherapy,^7,8^ but where certification (registration) is still not mandatory but highly recommended for higher standards as demanded by the public.^9^ Within the UK, this is a continuing debate, which we hope our survey will contribute to, in looking towards a new regulatory framework for clinical technologists as a whole, and dosimetrists in particular.^47^ A significant section of the UK dosimetrist workforce identified in our survey comes from HCPC registered therapeutic radiographers; a training pathway which brings its own distinctiveness and vital contribution to the tasks of the dosimetrist, alongside those training through other routes. Within the US, research has shown that there is no significant difference in standards between different training routes like these (when training for all dosimetrists is part of a professional framework),^48^ with department managers and supervisors having no reservations in employing from either stream.^48^

Our survey identified a high engagement with CPD and on-going training, a desire for more CPD but a need for more flexible/innovative ways of engaging with further education and training. Also clearly shown, was a willingness to engage publicly through shared learning and other methods, even in the face of challenges such as funding, time and workload demands in order to do so. There were similar themes identified within the literature; the need for innovative learning strategies (for many disciplines involved with radiotherapy) because of workload demands and lack of protected time^22,43,45^; like our survey, some key areas identified for further strengthening and development.^24^ Some noted the value of interprofessional learning,^25,29,34,35,43^ which has been proven elsewhere,^49^ and is indeed the ethos of multidisciplinary institutions like the BIR. We did not identify the length of service for our respondents, but the HERO initiative^13,14^ makes clear the need for long-term education and training needs across all disciplines, especially to help countries adopting newer technologies and techniques for the first time – something dosimetrists contribute to well,^44^ in ways which our survey identifies as our dosimetrist workforce would be willing to engage in, using flexible and innovative methods.

Somewhat disconcertingly, a sizeable proportion of our respondents (29%) indicated that they were not a member of a CPD scheme. This, coupled with free-text comments indicating the difficulties some dosimetrists have with undertaking CPD and, in some cases, lack of support and direction from managers and the career structure itself, would certainly lend further weight to the argument for having all dosimetrists (alongside clinical technologists) within a statutory register.^47^ As identified by the HCPC,^50^ CPD is ‘the way in which registrants continue to learn and develop throughout their careers so they keep their skills and knowledge up to date and are able to practice safely and effectively’.^50^ Statutory registration makes this a “must” and a “requirement”…so that registrants can practice safely and improve their practice, to the benefit of our patients. It would then be a requirement for all dosimetrists (not just those registered as therapeutic radiographers), and therefore a clear directive to managers and supervisors to not just encourage, but also enable CPD to happen within this staff group. Without CPD, one would not be able to stay registered….and one would not be able to practice clinically.

### Limitations

There are clear advantages and disadvantages to this form of convenience sampling approach within our survey. It had qualities of ease of distribution through known professional channels and contact lists like the Heads of Radiotherapy and Radiotherapy Physics (routes used by other surveys^18–20^ recently), and ease of accessibility through an electronic platform like SurveyMonkey in order to attract completion by individual dosimetrists across the UK. However, without specific targeting (such as through individual emails through a professional body or registration, as has been used elsewhere^10,24–27,29^ it is difficult to know aspects such as centre/regional responses (and whether the results are skewed to certain centres) or limit the risk of completion multiple times or by non-dosimetrists….although we feel there would be little to gain by individuals doing this. These are difficulties when this vital workforce does not all have statutory registration or a single professional body. Self-declared demographics would have helped and we will look to include some of these checks and balances in future work. Our approach, although, still brings in good, original data on characteristics and experiences of dosimetrists which will complement that of the IPEM^18^ and CoR^20^ surveys and help design CPD and development opportunities for this staff group, from their own experiences and situations.

### Futures

As mentioned earlier, our survey also captured a wealth of qualitative and free-text comments enriching the data presented here, especially in terms of working in developing areas such as specialised treatment planning, adaptive radiotherapy etc. In order to do these justice, the authors are looking to submit a separate follow-on publication on these. The work here should also act as a catalyst and platform for future work to run alongside that of our professional bodies, examining in more detail aspects such as workforce resource data, turnover, links between roles, bands and responsibility, and further details of job tasks (especially where dosimetrists work solely in areas outside treatment planning such as machine quality assurance and *in vivo* dosimetry). These could also continue to capture (as some of our free-text comments did) individual reflections on morale, stress and burnout and the effects on patient care, as captured recently by our clinical oncologist colleagues.^20^ Work is currently on-going through full, systematic literature reviews examining the differing titles, roles and responsibilities outside the UK, which should complement ESTRO-HERO work for dosimetrists elsewhere in Europe. More than anything, we hope this work (and follow-on publication) will give much greater insight into the dosimetrist workforce within the UK, especially for the provision of CPD and increased learning for improving the quality of patient care still further.

## Conclusions

Our survey is novel in approach and content, with results which have not been identified or published previously within the literature with regard to the UK dosimetrist workforce. Our quantitative results shared here identify the different training routes which are undertaken for working as dosimetrists in vital parts of the radiotherapy pathway, and the desire and direction of travel of many in terms of training and registration within the profession itself. The varied pattern of registration is clearly identified and contributes to the on-going discussions on the need for this for the important, public-facing role of dosimetrists. Job tasks are mainly based in computerised treatment planning, as one might expect; but spanning all complexities of modern radiotherapy techniques, with many undertaking other key tasks within the radiotherapy department; areas which could lend to role expansion and development in order to tackle workforce shortages across disciplines in radiotherapy. CPD is a crucial issue here where our survey identifies many engaged with it and the associated professional schemes for maintaining it; but almost 30% are not a member of a scheme – something statutory registration (*e.g.,* through HCPC) brings as a mandatory expectation and requirement in order to practise safely and effectively. There is a clear willingness and desire to be engaged in further CPD, to strengthen current practice and to develop new knowledge and skills, with all the benefits that that brings to our clinical practice in terms of safe and effective practice and benefit to service users – as defined by bodies such as the HCPC. There is a clear desire too, to go beyond this and engage publicly and with other disciplines in different and innovative ways of learning and teaching using modern digital methods. There is clear resonance with the literature in many of these aspects, emphasising that interprofessional and multidisciplinary working, learning and development (which includes dosimetrists) is a key requirement for developing safe and effective radiotherapy for the benefit of our patients.
